# Investigation of the anisotropic confinement-dependent brittleness of a Utah coal

**DOI:** 10.1007/s40789-020-00364-7

**Published:** 2020-09-21

**Authors:** Bo-Hyun Kim, Gabriel Walton, Mark K. Larson, Steve Berry

**Affiliations:** 1 CDC/NIOSH, 315 E. Montgomery Ave., Spokane, WA, USA; 2 Department of Geology and Geological Engineering, Colorado School of Mines, Golden, CO, USA; 3 Department of Geological Engineering, Montana Tech, Butte, MT, USA

**Keywords:** Utah coal, Spalling limit, AUCS/UCS ratio, Damage characteristic, Post-yield dilatancy, Anisotropic

## Abstract

Changes of failure mechanism with increasing confinement, from extensional to shear-dominated failure, are widely observed in the rupture of intact specimens at the laboratory scale and in rock masses. In an analysis published in 2018, both unconfined and triaxial compressive tests were conducted to investigate the strength characteristics of 84 specimens of a Utah coal, including the spalling limits, the ratio of apparent unconfined compressive strength to unconfined compressive strength (UCS), the damage characteristics, and the post-yield dilatancy. These mechanical characteristics were found to be strongly anisotropic as a function of the orientation of the cleats relative to the loading direction, defined as the included angle. A total of four different included angles were used in the work performed in 2018. The authors found that the degree of anisotropic strength differed according to the included angle. However, the transition from extensional to shear failure at the given confinements was not clearly identified. In this study, a total of 20 specimens were additionally prepared from the same coal sample used in the previous study and then tested under both unconfined and triaxial compressive conditions. Because the authors already knew the most contrasting cases of the included angles from the previous work using the four included angles, they chose only two of the included angles (0° and 30°) for this study. For the triaxial compressive tests, a greater confining stress than the mean UCS was applied to the specimens in an attempt to identify the brittle-ductile transition of the coal. The new results have been compiled with the previous results in order to re-evaluate the confinement-dependency of the coal behavior. Additionally, the different confining stresses are used as analogs for different width-to-height (*W*/*H*) conditions of pillar strength. Although the *W*/*H* ratios of the specimens were not directly considered during testing, the equivalent *W*/*H* ratios of a pillar as a function of the confining stresses were estimated using an existing empirical solution. According to this relationship, the *W*/*H* at which in situ pillar behavior would be expected to transition from brittle to ductile is identified.

## 1 Introduction

High stress environments bring many technical challenges in deep underground mining. With the continued increase of mining depth, a number of new challenges have been encountered in underground excavations (Kaiser et al. 2011). Dynamic failure events (also called “bumps” or “bursts”) have been documented for well over 100 years within the American underground coal mining industry. The assessment of such dynamic failure hazards in coal mines depends on fundamental knowledge of coal mechanical behavior. A robust characterization of the confinement-dependent mechanical behavior of coal is of importance in order to mitigate the hazards potentially threatening mine workers (Kim et al. 2018a, b).

Given that coal is expected to fail in a brittle manner under most practical mining conditions, behavioral features associated with its relatively low tensile strength, such as the transition from extensional to shear failure as a function of confining stress, have to be considered and reflected in the adopted failure criteria. In particular, rock failure in tension takes place at low confinement around excavations due to brittle cracking at the grain scale (Diederichs 2007). The prospect of extensional failure diminishes as the confinement increases away from the excavation boundary. Therefore, it is anticipated that the transition from an extensional to a shear failure mechanism occurs as the confinement level changes and the conditions for tensile failure are prevented or strongly diminished, such as is expected in the core of a coal pillar (Kaiser et al. 2011). The horizontal stresses inside a pillar increase with an increase in the width-to-height (*W*/*H*) ratio (Bieniawski 1968b). Accordingly, the effect of confining stress on laboratory compressive strength can be used as an analog for the effect of W/H ratio on pillar strength (Moomivand and Vutukuri 1996).

The exact nature of failure mechanism transitions is likely to be influenced by the highly anisotropic characteristics of coal seams associated with geologic structure and mining-induced spatial redistribution of stress in coal pillars (Kim et al. 2018b). Kim et al. (2018b) investigated the strength, brittleness, and dilation anisotropy of a Utah coal using both unconfined and triaxial compressive tests by considering four orientations between the cleat and the axial loading direction in the coal specimen. From the investigation, the mechanical characteristics were found to be highly anisotropic and controlled by the orientation of the cleats relative to the loading direction. However, the transition from extensional to shear failure at the given confinements was not clearly observed as anticipated based on the s-shaped brittle-failure criterion (Kim et al. 2018b). This criterion has clear divisions between extensional failure, transitional failure, and shear failure, and it was our objective to use this criterion to model the coal strength.

In order to study the strength characteristics of coal under high confinement and, hence, shed insights for pillar stability and design, a second laboratory testing campaign was conducted to examine the effect of confinement on the transition of the failure mode of the Utah coal. A total of 20 additional specimens were recovered from the Utah coal sample that was used in the previous study (Kim et al. 2018b). In that study, all confining stresses were no larger than 50% of the mean unconfined compressive strength. For the present study, a higher magnitude of confining stress than the originally determined mean unconfined compressive strength (UCS) was applied to the specimens. Because in the previous study the included angle (the angle between the cleat and the loading direction) was found to be an important variable affecting the coal strength (Kim et al. 2018b), in this study the two most contrasting cases of the included angle, 0° and 30°, were used to evaluate the mechanical behavior of the coal under high confinement. The confining stress as a ratio of the mean UCS was increased in successive tests until a relatively ductile post-peak stress–strain behavior stage was achieved. The maximum applied confining stress was equivalent to 5 times (for 0° of the included angle) and 4 times (for 30° of the included angle) the mean UCS values for the two cases.

## 2 Laboratory tests of the coal specimens considering high confinements

For this study, 14 triaxial tests and 6 UCS tests were performed at Montana Tech in Butte, MT, USA. The purpose of the triaxial tests was to examine the characteristics of brittle/ductile transition in coal, and the UCS tests were used to confirm consistency and comparability with the results from the previous study conducted by Kim et al. (2018b). A total of 20 cylindrical specimens were cored from the same Utah coal boulders from which the cores in the previous study were obtained and then tested by Kim et al. (2018b). The coal boulders were collected from the Tank seam of the Blackhawk Formation, Wasatch Plateau, Central Utah. Because the influence of the included angle between the cleats and the loading direction was already studied in detail previously (Kim et al. 2018b), coring was conducted so that only the two most contrasting cases of the included angle with respect to mechanical behavior (0° and 30°) were obtained and prepared for the tests. Using the same procedures adopted by Kim et al. (2018b), all specimens in this study were cored to an approximate diameter of 44 mm and cut to be within the ASTM (D 4543-08) recommended range of 2.0 to 2.5 length-to-diameter ratio. The ends of the specimens were ground on a surface grinder to be within the ASTM (D 4543-08) flatness tolerance of 0.025 mm. Specimen end parallelism and perpendicularity tolerances were verified using a parallelism testing gauge.

**Fig. 1:**
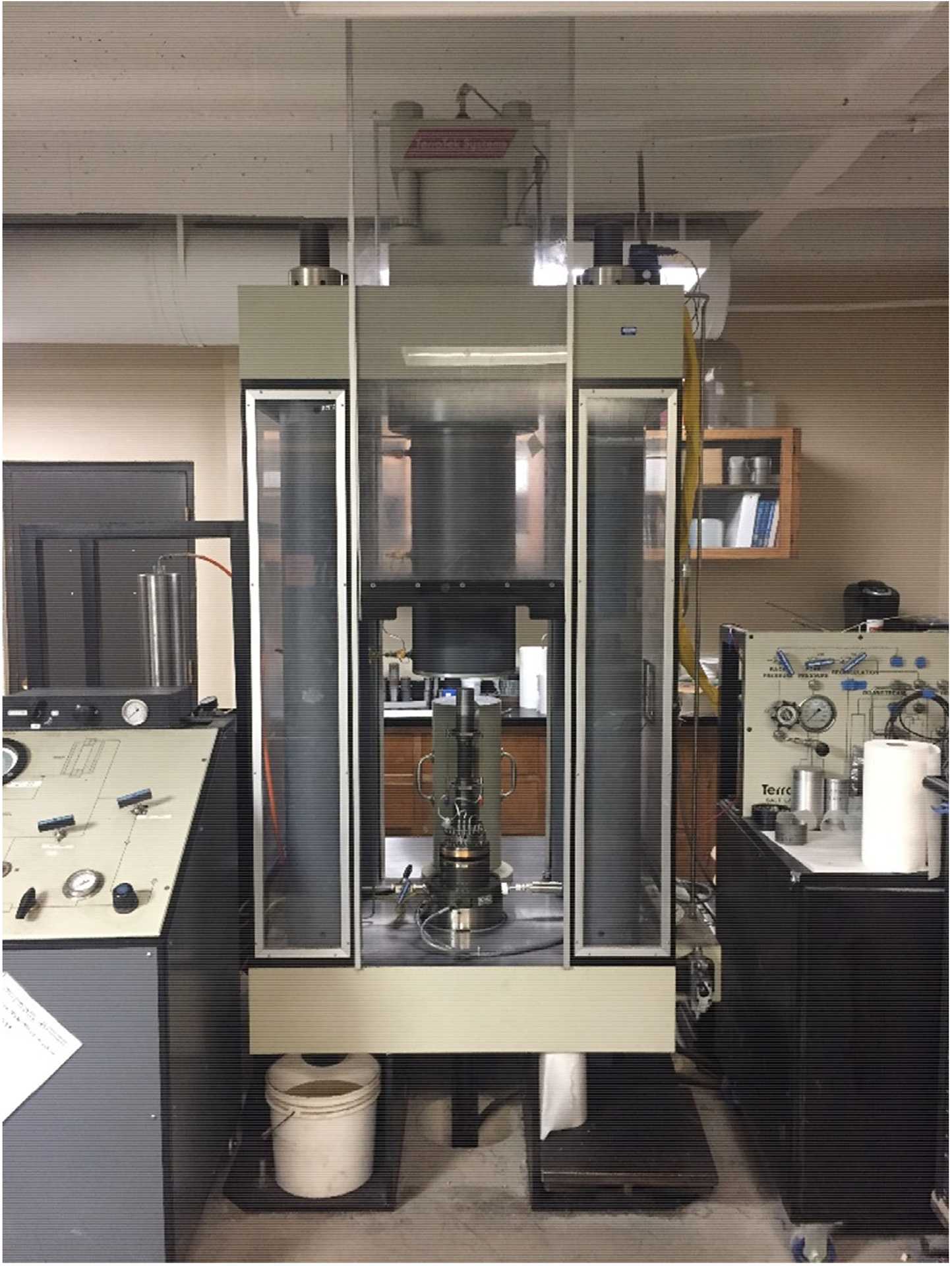
Triaxial test machine at Montana Tech used for testing the coal specimens

The tests were performed using a TerraTek Model FX-S-33090 closed loop digital servo controlled load frame. The testing system, shown in Fig. 1, consists of a TerraTek 113.4 tonnes-force load cell to measure force, two impervious endcaps, two Schaevitz MHR 500 LVDTs to measure axial displacement, a TerraTek radial cantilever transducer to measure lateral strain, and spherical seats to minimize the risk of non-uniform loading of the specimen.

The 14 triaxial samples were jacketed with 0.5-mm-thick Dunbar-1635F flexible 2:1 Polyolefin to prevent the confining fluid from penetrating the sample. The six UCS samples were tested without jackets. Both the UCS and the triaxial test procedures followed the ASTM suggested method (D7012). Both the triaxial and UCS test procedures were run under axial strain rate control with specimens axially loaded at a strain rate of 2.54 × 10^−5^/s.

## 3 Analysis of the test

### 3.1 Observation of coal specimen failure mode and its interpretation using the S-shape failure criterion

Kaiser and Kim (2008a, 2015) introduced an S-shaped criterion for brittle rock strength as shown in Eqs. (1)–(3). The S-shaped criterion is presented here to illustrate the fact that strength in the low-confinement zone is disproportionally lower than in the high-confinement zone by an even greater margin than typically predicted by more conventional (shear) strength models (i.e. Hoek–Brown). This phenomenon is the result of changes in the failure mode for brittle rocks, with extensional failure occurring at low confinement and shear failure occurring at high confinement.

(1) $$\sigma_{1}^{\prime }=k_{2}\sigma_{3}^{\prime }+\text{AUCS}+\left[\frac{\left(\text{UCS}-\text{AUCS}\right)}{1+e^{\left(\sigma_{3}^{\prime }-\frac{\sigma_{3}^{0}}{\delta \sigma_{3}}\right)}}\right]$$

where UCS, the lower *y*-intercept, is the mean unconfined compressive strength obtained from the laboratory test; AUCS is the *y*-intercept apparent UCS obtained from the linear back projection of a linear fit to high confinement data; and *k*_2_ is the gradient of this high confinement linear fit. Therefore, the mean UCS represents intact rock strength at zero confinement, whereas the AUCS can be estimated as the unconfined component of intact rock strength (i.e. analogous to cohesion) at high confinement (Kaiser and Kim 2008a, 2015).

The transition curve from the lower confinement region (extensional failure) to the higher confinement region (shear failure) is called the spalling limit and is assumed to start at the origin with a slope of *k*_s_ = *σ*_1_/*σ*_3_ (Kaiser and Kim 2008a, 2015).


(2) $$\sigma_{3}^{0}=\frac{\left(\text{UCS}-\text{AUCS}\right)}{2\left(k_{\text{s}}^{\prime }-k_{2}\right)},\left(k_{\text{s}}=\frac{\sigma_{1}^{\prime }}{\sigma_{3}^{\prime }}\right)$$

(3) $$\delta \sigma_{3}=C\sigma_{3}^{0},(C=0.1∼0.3)$$

Kim et al. (2018b) did not clearly observe the transition from extensional to shear failure in specimen failures at the confinements originally considered during testing. The authors presume that this transition was not observed because the original confining stresses as a ratio of the mean UCS (i.e., 1%, 2%, 10% or 50%) used by Kim et al. (2018b) might not be suitable to fully characterize the strength characteristics of the coal specimens. Although (Hoek 2007) suggested that the confinement range considered should be at least up to one-half of the mean UCS of a rock, this was based primarily on data from rocks much less brittle than coal, where extensional failures are more easily suppressed by lower levels of confinement.

**Fig. 2:**
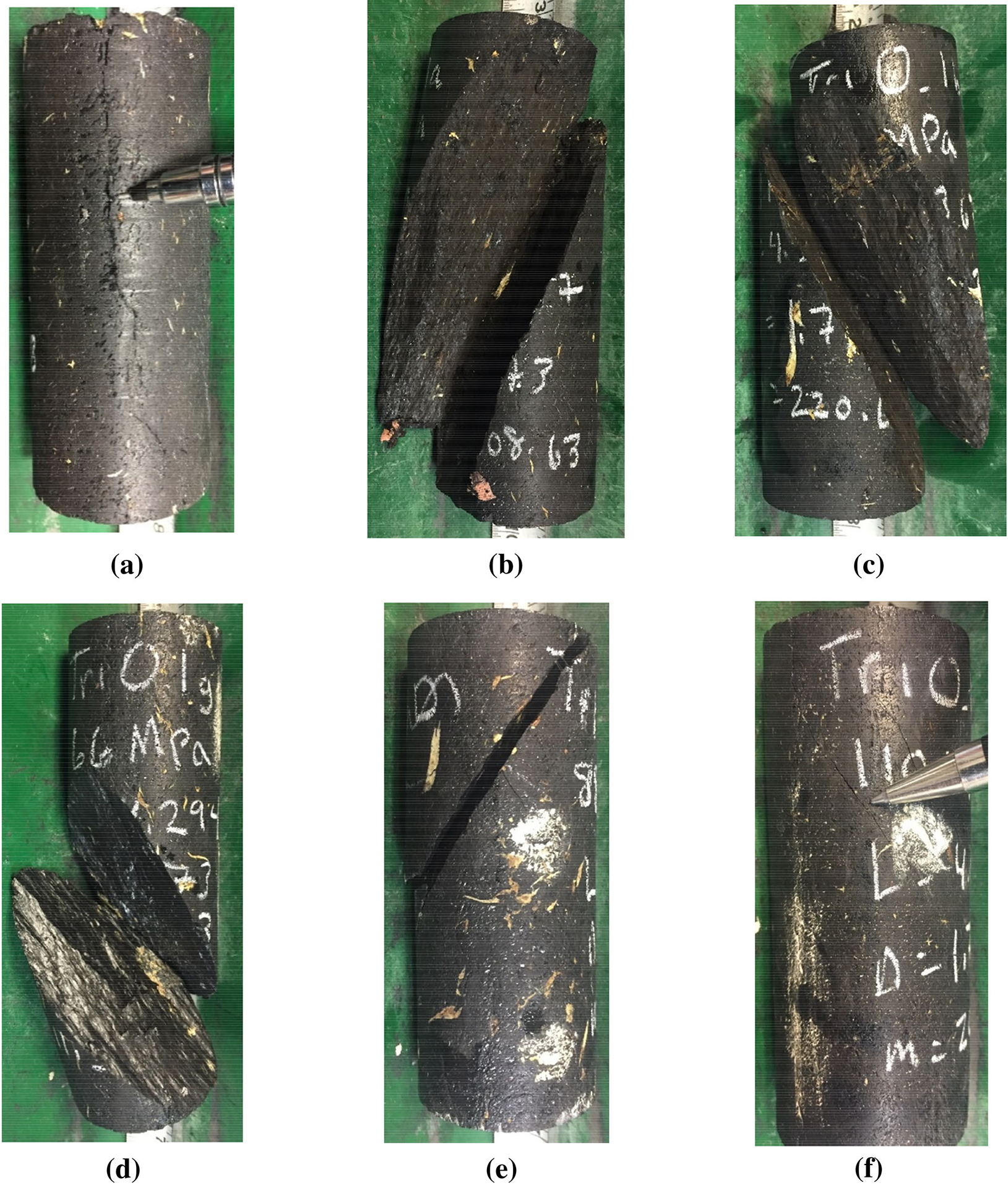
Examples of failed coal specimens with 0° included angle (mean UCS 22 MPa) after triaxial compressive strength testing: **a** confining stress of 11 MPa; **b** confining stress of 22 MPa; **c** confining stress of 44 MPa; **d** confining stress of 66 MPa; **e** confining stress of 88 MPa; **f** confining stress of 110 MPa)

**Fig. 3:**
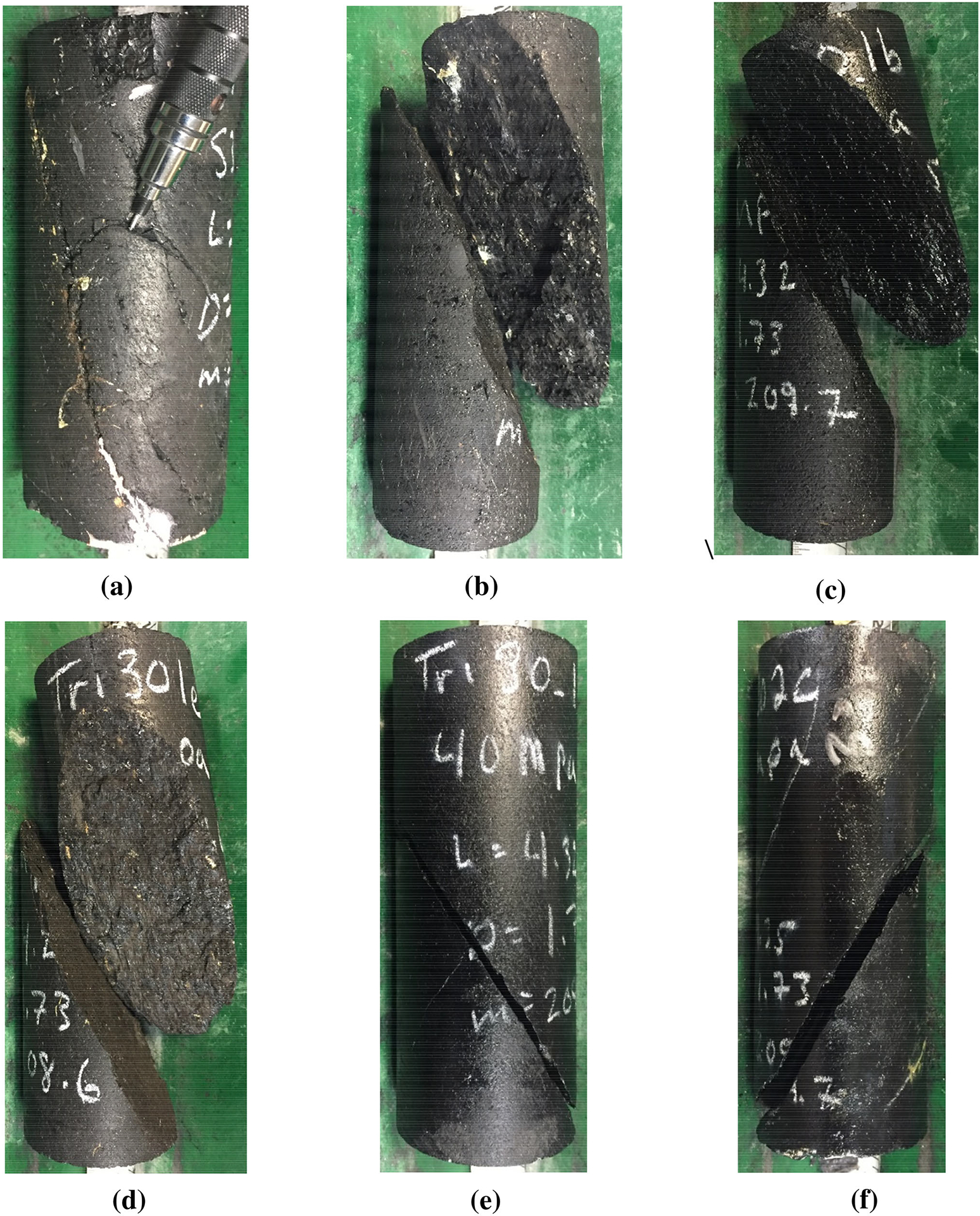
Examples of failed coal specimens with 30° included angle (mean UCS 16 MPa) after triaxial compressive strength testing: **a** confining stress of 4 MPa; **b** confining stress of 8 MPa; **c** confining stress of 16 MPa; **d** confining stress of 32 MPa; **e** confining stress of 40 MPa; **f** confining stress of 64 MPa)

The photographs obtained after the new suite of tests show that all the specimens failed mainly in shear when the confining stress was equal to or greater than the mean UCS for the included angle of 0° and equal to or greater than twice the mean UCS for the included angle of 30° (see Figs. 2, 3). For the 0° included angle specimens, at 11 MPa confinement (Fig. 2a), extensional-axial cracking was observed. With increasing confinement, brittle shear failure was observed. Figure 2f shows a shear failure surface typically associated with semi-ductile failure at the highest confining stress (110 MPa). At that strain, localization is still occurring (cracks visible), but it is not well defined or limited to a single shear plane. In fact, this result is comparable to the failure pattern of a naturally more ductile rock, such as Indiana Limestone under 15 MPa of confining stress (Walton et al. 2017).

Kaiser and Kim (2008a, b) implied that the ratio (as a percentage) of confining stress to UCS at to shift from extensional failure to transitional failure is less than 10%, and that the ratio at the shift from extensional to shear failure is around 20%. Based on the observations from the photos in Figs. 2 and 3, one would expect the boundaries in coal to be closer to at least 50% for the shift from extensional failure to transitional failure33. Additionally, one would expect the shift from transitional failure to shear failure to occur near 100% of the mean UCS and the shift to fully ductile shear to occur at confinements greater than 100% of the mean UCS for these coal specimens. The discrepancy between these confinements and those proposed by Kaiser and Kim (2008a, b) may have occurred because the original thresholds were determined based on hard rock types with a lower brittleness-to-stiffness and brittleness-to-strength ratios than those of coal and without the strongly anisotropic nature of coal. These modified confinement levels interpreted for the different failure modes for the coal in question are as shown in Table 1.

**Fig. 4:**
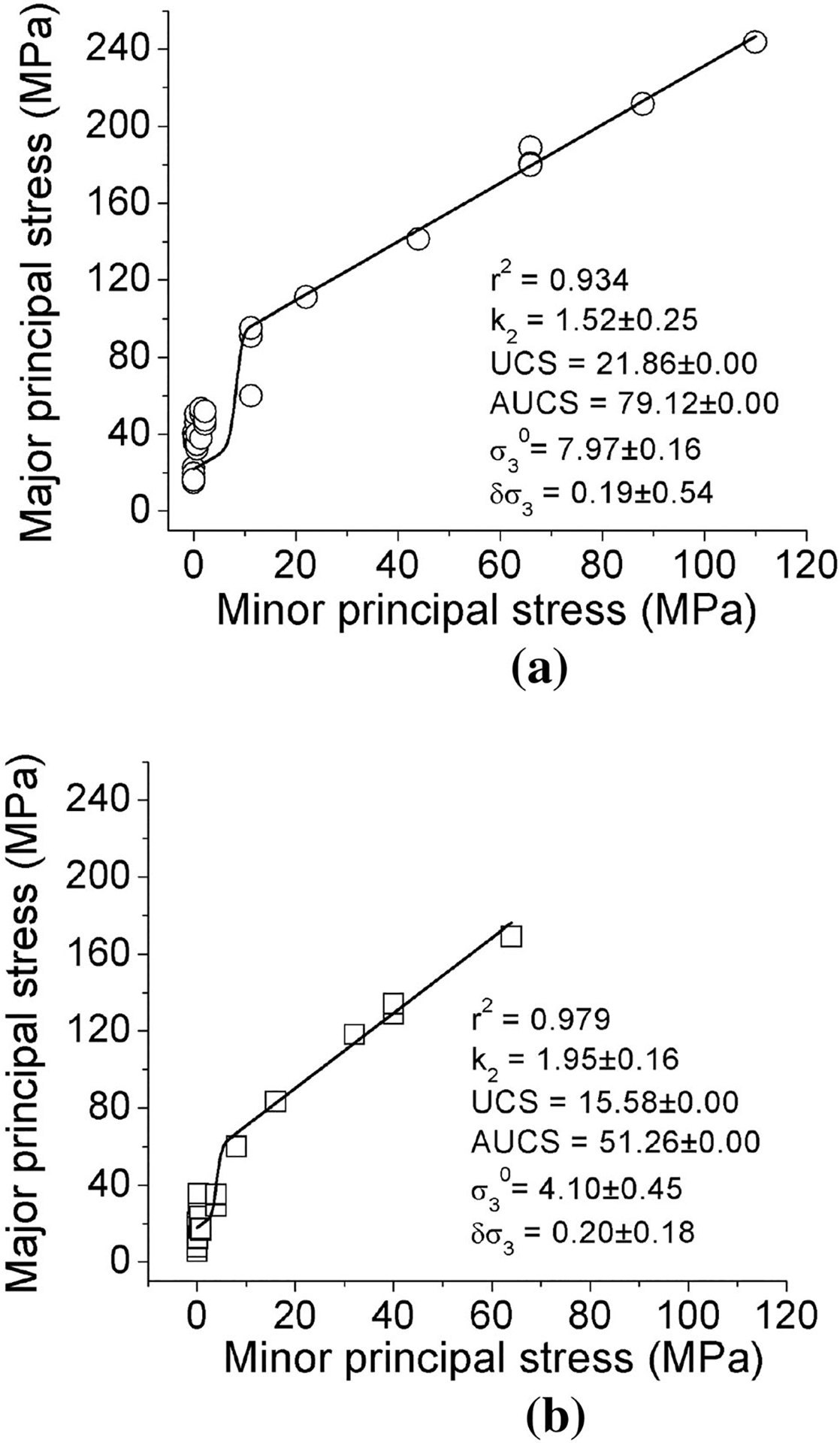
Results of triaxial compressive tests (**a** 0° included angle and **b** 30° included angle) fitted to s-shaped brittle failure criterion

Curves were fit to the laboratory testing data using the Eqs. (1)–(3) to produce the results presented in Fig. 4. These plots were generated by adding the new data obtained in this study to the previous data presented by Kim et al. (2018b). The results of fitting the s-shaped criterion to data from the specimens with an included angle of 0° and 30°, respectively, are shown. It is evident that the data can be represented adequately well (*r*^2^ = 0.93 and *r*^2^ = 0.98) by an s-shaped failure criterion.

The UCS near excavation walls and apparent compressive strength (AUCS) in low-confinement conditions are typically encountered at a distance of less than one radius from the excavation wall (Kaiser et al. 2011). UCS is the lower y-intercept of the formula, or the unconfined compressive strength obtained from the laboratory test; AUCS is the y-intercept obtained from the back-projection of a linear fit to high confinement data. It is observed that the AUCS of the coal was found to be over twice greater than the UCS if the applied confinement to the coal was as much as 10% of the UCS (Kim et al. 2018b). The spalling limits and the ratio of AUCS to UCS for the different conditions of the included angle are presented in Table 2. The values of the spalling limit estimated in this study were slightly lower than the spalling limit of an Australian coal at 38 (Buzzi et al. 2014), of a Chinese coal at 40 (Gao and Kang 2016) and of the previous work by Kim et al. (2018b) at 41 to 57.

The difference in the behavior of this coal compared to harder intact rock and the different AUCS and spalling limits obtained by this modification in procedure suggests that accurate simulation of a coal pillar with a numerical model must be done with a carefully determined material model and material properties. A lesson learned here is that if the confinement level associated with failure mode in the s-shaped criterion is not carefully chosen for characterizing a highly brittle coal, the spalling limit would be overestimated but the ratio of AUCS to UCS (confined strength) would be underestimated.

### 3.2 Effect of confinement on post-peak (brittle/non-brittle) behavior in the coal specimens

This section discusses the results of the laboratory tests in order to investigate the post-peak behavior as a function of the critical included angles in the coal specimens. It is very common for triaxial tests of coal specimens to show evidence of shear failure at all confining stress levels, including under unconfined conditions. However, according to the s-shaped brittle failure criterion (Kaiser and Kim 2008a, 2015; Kim et al. 2018b), there are mechanistic divisions between extensional failure, transitional failure, and shear failure as a function of confining stress. Evidence of a failure plane is often present in the final state of the specimen, but may not be representative of the failure process just after the peak strength is achieved.

**Fig. 5:**
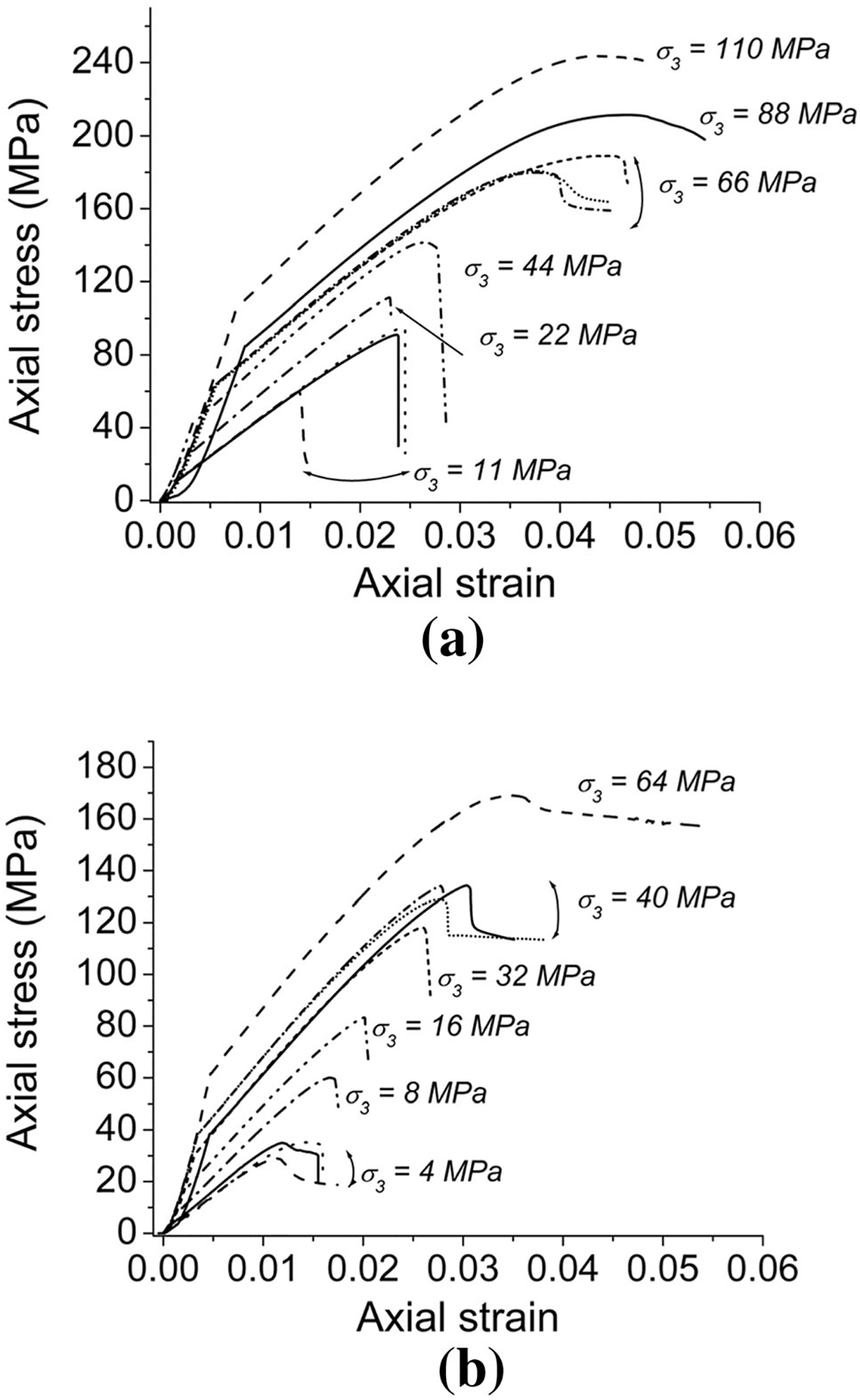
Complete axial stress–strain curves obtained in triaxial compression tests on the coal at various confining stresses **a** 0° of the included angle; **b** 30° of the included angle

Cook (1965) and Cook and Hojem (1966) noted that a laboratory test specimen may crush violently or benignly, depending on the stiffness of the testing system relative to the post-failure stiffness of the specimen. In the transitional failure regime, episodes of brittle response from microcracking can cause small drops in load. A test system with insufficiently high stiffness might then apply dynamic forces which drive more fracturing than would a very stiff testing system. For this reason, a very stiff testing machine, shown in Fig. 1, was employed for this study. The stress-versus-strain curves obtained (Fig. 5) are considered reliable because any parts of the curves that were not densely represented by points (i.e. where control was lost due to brittle failure at peak stress, or during the unloading segments after the completion of the test) have been removed. It should be noted that very large amounts of axial strain were applied in order to reach peak strength, with the large strains accommodated by localized displacements along pre-existing discontinuities, resulting in the localized shear failures observed even in specimens subjected to confining stresses well above the UCS and displaying semi-ductile stress–strain behavior. The localized shear failures observed are in agreement with those observed in the previous study (Kim et al. 2018b).

Figure 5 shows the stress–strain curves of the triaxial compressive tests on the 14 coal specimens conducted with different confining stresses. Figure 5a presents the results of tests on the specimens with an included angle of 0°. Figure 5b illustrates the test results on the specimens with an included angle of 30°. The confinements of 11 MPa for 0° and 4 MPa for 30° are obtained from the study performed by Kim et al. (2018b). The confining stresses of 22 MPa and 16 MPa are equivalent to the mean UCS for the included angles of 0° and 30°, respectively. For the specimens having an included angle of 0° as shown in Fig. 5a, the results show that following attainment of peak strength, the stress–strain curves show strain-weakening (semi-ductile behavior) when the applied confining stress was 66 MPa. This pressure is as high as 3 times the mean UCS. For the specimens having an included angle of 30° as shown in Fig. 5b, the results show a similar semi-ductile residual stress–strain curve when the applied confining stress was 40 MPa. This pressure is as high as 2.5 times the mean UCS. Even at the highest confining stresses in both cases, the stress–strain data indicate that perfectly plastic behavior was not attained. This is consistent with the fact that for all tests, observed shear failure was localized rather than diffuse.

## 4 Damage characteristics and dilatancy of the coal specimens

Standard methods to evaluate specimen brittleness include evaluation of post-peak stress–strain data and visual inspection of failure mode and strain localization patterns. The exact failure patterns visually observed in specimens are a function of the pre-existing flaws and planes of weakness in the coal and the large amount of strain applied prior to reaching failure, but may also depend to some extent on the nature of the post-peak loading applied to the specimens (e.g. machine stiffness and control mechanism, amount of post-peak strain applied, etc.). The fitting of the s-shaped criterion to peak strength data does provide some information on failure mechanisms of specimens at different confining stresses. Although fitting the S-shaped criterion can help put a lower bound on the confining stress at which the brittle-ductile transition occurs (because only shear failures in rock can be ductile in nature), such fitting cannot help to delineate between brittle and ductile failures. Accordingly, it is valuable to identify pre-peak indicators of the ductility of the inelastic specimen deformation mechanisms occurring in samples loaded under different confinement conditions.

Previously, Walton et al. (2017) identified such a pre-peak indicator of deformation mechanism ductility using a database of post-peak Indiana Limestone compression tests. In particular, they found that the confinement level at which the principal stress trend corresponding to the reversal of the inelastic volumetric strain curve (a.k.a. Crack Volumetric Strain Reversal—CVSR; (Eberhardt et al. 1998)) increases in slope represents the onset of ductile behavior. Conversely, the confinement level at which the CVSR stress becomes coincident with the peak stress represents the onset of fully ductile (or even strain-hardening) behavior. The mechanistic significance of the CVSR stress is that it corresponds to the stress at which extensile microcracks begin to form within a rock specimen (Diederichs and Martin 2010; Hoek and Martin 2014). As the failure mode transitions from macroscopically extensile to macroscopically shear-based with a brittle failure plane defined by the coalescence of extensile microcracks, the CVSR stress maintains its mechanistic significance. As confinement is increased even further, extensile microcrack growth is suppressed, and semi-ductile or fully ductile mechanisms dominate deformation, meaning the CVSR stress ceases to relate to extensile crack formation (Walton et al. 2014). This change in the nature of the CVSR stress corresponds to the point at which the slope of the CVSR principal stress trend increases. While the coal presented in this work is naturally much more brittle than Indiana Limestone at any given confinement, the Indiana Limestone still behaved in a highly brittle manner under unconfined conditions; accordingly, when using the brittle-ductile transition framework established for Indiana Limestone, it must be recognized that the same transitions will occur in coal at significantly higher confining stresses.

**Fig. 6:**
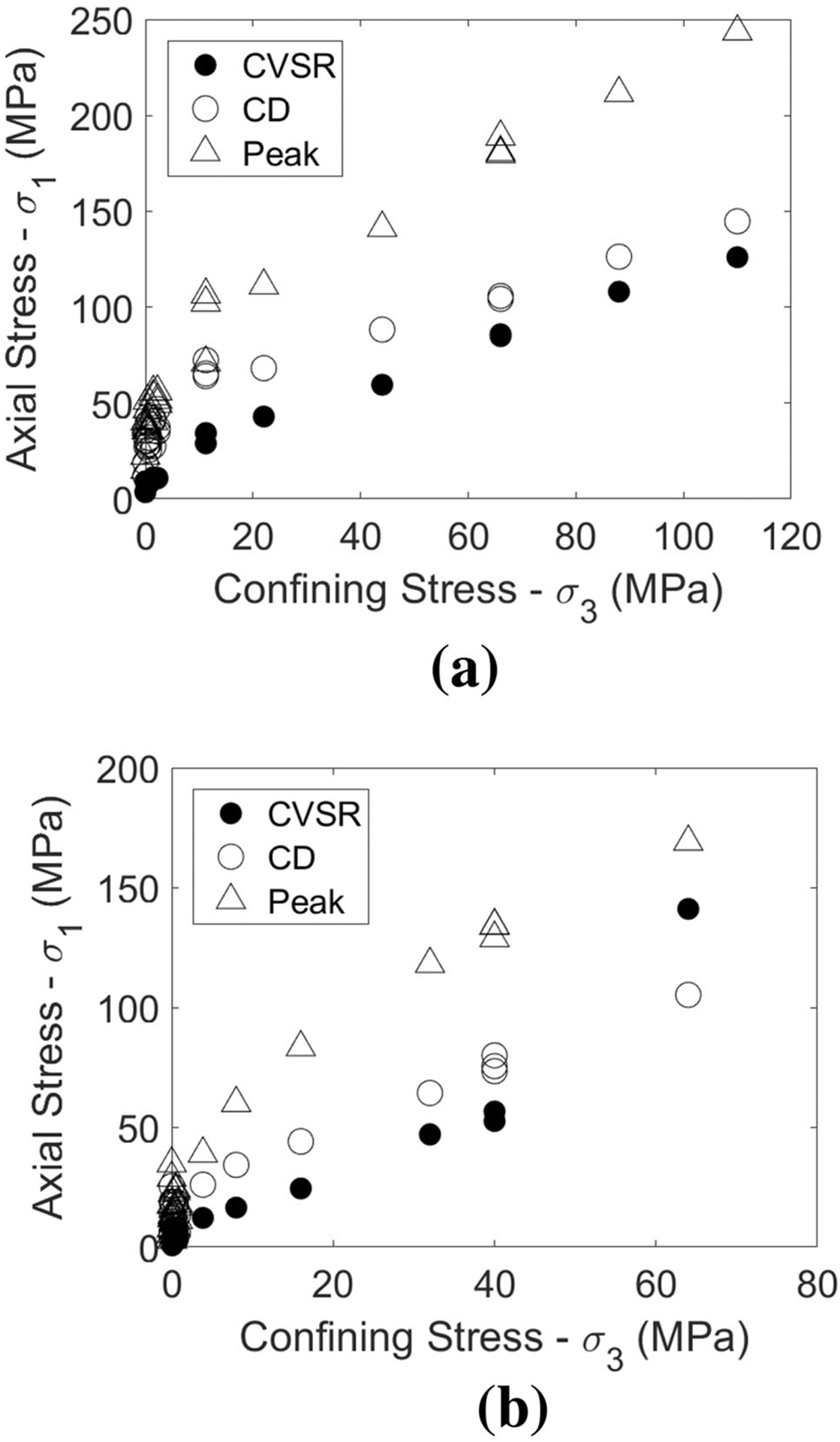
Crack Volumetric Strain Reversal stress, Crack Damage stress (CD), and peak strength data for 0° included angle (**a**) and 30° included angle (**b**) Note that the crack damage stress was determined as the point of axial strain non-linearity using the tangent modulus reversal method (Eberhardt 1998; Walton et al. 2015, 2017)

In the case of the coal considered in this study, the CVSR data did show an apparent increase in slope for the 30° included angle samples. This change in slope occurred between 40 and 64 MPa of confining stress (see Fig. 6). The fact that a change in slope was observed, but that the CVSR stress for 64 MPa of confining stress is not coincident with the peak stress suggests that the onset of semi-ductile behavior for the 30° included angle specimens is between 40 and 64 MPa of confinement, and that the onset of fully ductile behavior is beyond 64 MPa of confinement. This conclusion is broadly consistent with the available stress–strain data as shown in Fig. 5.

The 0° included angle data are somewhat less conclusive. Walton et al. (2014) found that the CVSR stress for fully ductile specimens consistently matched the peak stress. Accordingly, the lack of coincidence between the CVSR stress and the peak stress for any of the tests indicates that the onset of fully ductile behavior in this case is beyond 110 MPa of confinement. The lack of a change in CVSR stress slope, however, is not indicative that semi-ductile behavior has not been achieved. Walton et al. (2014) found that in the range of confinements where semi-ductile behavior was observed, the CVSR data were erratic, with some sample data following the lower confinement trend and some data with higher values indicative of the CVSR stress slope change. Based on all the available data, the most plausible explanation is that the onset of semi-ductile behavior occurs near a confinement of 110 MPa (as indicated by the relatively gentle post-peak stress–strain curve slope shown in Fig. 5 and the multiple zones of ductile strain localization shown in Fig. 2), and that the CVSR value for the one *σ*_3_ = 110 MPa sample tested happened to be one of the cases that followed the lower confinement trend.

Another phenomenon that occurs prior to the attainment of peak strength that can be used to assess the brittleness or ductility of a specimen’s behavior is inelastic volume change. The onset of inelastic volumetric change is typically roughly coincident with the onset of non-linearity in the axial stress—lateral strain curve. Since non-linear stress–strain behavior initiates at stresses below the peak strength, especially for high confinement conditions (see Fig. 4), volume change information indicative of sample brittleness can be obtained easily from pre-peak stress–strain data.

It is understood that volumetric dilatancy (volume expansion during yield) is typically associated with brittle deformation mechanisms (crack formation and opening), whereas constant volume (zero dilation) “flow” typically occurs when ductile shear is the dominant deformation mechanism (Cook 1970; Hill 1950; Walton 2014; Walton et al. 2017). Empirically, the relationship between dilatancy and brittleness has been documented for several rocks in terms of the peak dilation angle and the Hoek–Brown parameter (*m*_i_) (Walton 2019).

Walton et al. (2017) proposed a conceptual model for changes in dilatancy across the brittle-ductile transition. In particular, they found the following: (1) Samples with brittle behavior displayed the highest peak dilation angles and showed a significant decrease in dilation angle after the attainment of peak dilation angle as a function of plastic shear strain; (2) Samples with semi-ductile behavior displayed lower peak dilation angles (typically on the order of 1/6 to 1/3 the peak dilation angle under unconfined conditions) and relatively flat dilation angle trends as a function of plastic shear strain; (3) Samples with fully ductile behavior displayed dilation angles tending to vary around 0° (typically starting with some negative dilation angle values, indicating initial compactant behavior), and attainment of the peak dilation angle was delayed until very large values of plastic shear strain are reached (on the order of 6 to 10 times the values at which peak dilation angle was attained for brittle samples). With these bench-marks as a point of reference, one can now examine the dilation angle data obtained for the coal currently being studied.

**Fig. 7:**
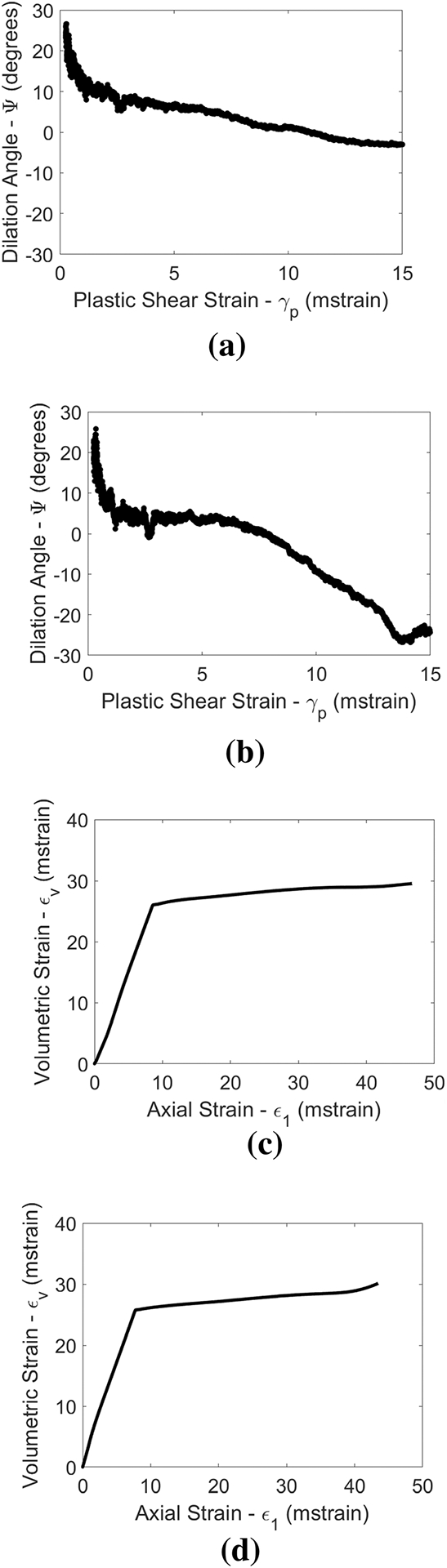
Dilation angles as a function of plastic shear strain (top) and volumetric-axial strain plots (bottom) for 0° included angle specimens tested at 88 MPa (left) and 110 MPa (right) of confining stress

For the 0° included angle data, the dilation angle data (see Fig. 7) are consistent with more ductile behavior of the 110 MPa confinement sample than the 88 MPa confinement sample. This is because of the relatively lower dilation angles for this sample at all plastic shear strains, and the relative prominence of compactant behavior (negative dilation angles). Note that the compactant behavior in the case of the 110 MPa confinement sample appears in the volumetric strain versus axial strain plot (also shown in Fig. 7) as an increase in slope beyond around 40 m strain (positive volumetric strain increments correspond to volumetric contraction). Generally speaking, both of these data sets are consistent with the semi-ductile volumetric behavior described by Walton et al. (2017) for Indiana Limestone (albeit at much lower confining stresses). Although the 110 MPa confinement sample behavior is relatively ductile from a volumetric deformation stand-point, it cannot be classified as fully ductile, since the dilation angle is not initially negative, and the peak dilation angle is attained almost immediately; if the behavior were truly ductile, one would expect the peak dilation angle to be attained at very large plastic strain values.

**Fig. 8:**
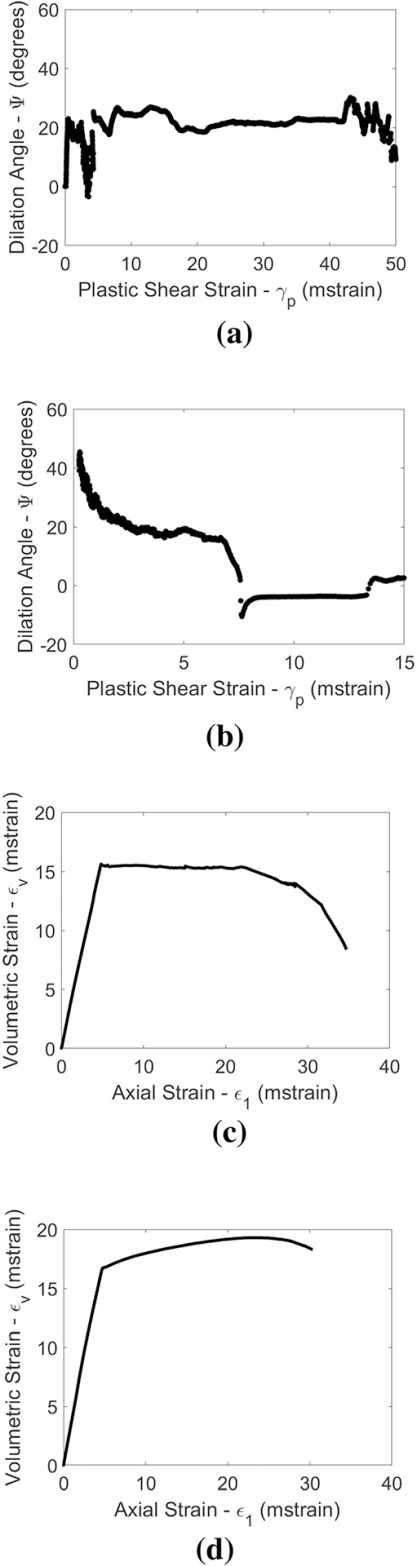
Dilation angles as a function of plastic shear strain (top) and volumetric-axial strain plots (bottom) for 30° included angle specimens tested at 40 MPa (left) and 64 MPa (right) of confining stress

For the 30° included angle data, the dilation angle data (Fig. 8) generally indicate more brittle behavior than that of the 0° included angle samples. Both show primarily dilatant specimen behavior (although there are some small negative dilation angles in the case of the 64 MPa confinement sample). The 40 MPa confinement sample volumetric behavior is indicative of somewhat brittle behavior (fully dilatant volumetric behavior over a large range of strains), whereas the 64 MPa confinement sample volumetric behavior could be considered consistent with the brittle-ductile transition. Note, however, that the volumetric behavior of the 64 MPa confinement 30° included angle is still slightly more brittle than that of the 88 MPa confinement 0° included angle data (higher peak dilation angle and actual reversal of the volumetric-axial strain curve is observed).

## 5 Discussion

Overall, the post-peak stress–strain data, post-failure specimen photos, CVSR data, and dilation angle data all provide consistent indications of the confining stress ranges over which the brittle-ductile transition occurs in the tested coal:

1. For the 0° included angle samples, it appears that the transition to semi-ductile behavior initiates around *σ*_3_ = 88 MPa (where *σ*_1_/*σ*_3_ at peak strength is approximately 2.4), and that at *σ*_3_ = 110 MPa (where *σ*_1_/*σ*_3_ at peak strength is approximately 2.2), the specimen behavior is almost fully ductile; in other words, brittle-ductile transition should occur at a confining stress slightly above 110 MPa (i.e. at a *σ*_1_/*σ*_3_ slightly less than 2.2).
2. For the 30° included angle samples, it appears that the transition to semi-ductile behavior initiates at confining stresses just around *σ*_3_ = 40–64 MPa (where *σ*_1_/*σ*_3_ at peak strength is 2.6–3.2), suggesting fully ductile behavior should occur for confining stresses somewhat higher than 64 MPa (i.e. at a *σ*_1_/*σ*_3_ ratio less than 2.6).

Although these findings could be refined by conducting more tests (both at the confinements already considered as well as at higher confining stresses), these results provide an initial basis for comparison with prior findings on the brittle-ductile transition in rocks (Mathey and Van der Merwe 2016).

Perhaps the most significant work on the brittle-ductile transition in rocks was that of Mogi (1966) where he introduced what is now known as “Mogi’s Line”: the line of slope 3.4 in *σ*_1_ − *σ*_3_ space that delineates a boundary between ductile and brittle behavior of rocks. In other words, he found that for most rocks (porous and non-porous, igneous, sedimentary, and metamorphic), when the peak strength to confinement ratio for a specimen was less than 3.4, the specimen would consistently display ductile deformation behavior. Inherent in this delineation of the brittle-ductile transition is the assumption that the brittleness of rocks is strongly correlated with their strength; this assumption is generally valid, as stronger rocks do indeed tend be more brittle (Mathey and Van der Merwe 2016), therefore requiring a higher *σ*_3_ to reach the brittle-ductile transition and the critical *σ*_1_/*σ*_3_ ratio of 3.4. Some rock types, however, are inherently more or less brittle than other rocks with similar strengths. Mogi (1966) noted this, and defined a second line with a slope of *σ*_1_/*σ*_3_ ≈ 5 to delineate the brittle-ductile transition for carbonate rocks, which are often composed almost exclusively of the highly ductile mineral calcite.

In considering coal, it is not surprising that the brittle-ductile transition occurs at *σ*_1_/*σ*_3_ ratios below 3.4. Contrary to carbonate rocks, coal is highly brittle relative to its strength (Mathey and Van der Merwe 2016). Let us consider the Hoek–Brown curve fit parameter, *m*_i_, which is approximately equal to the ratio of unconfined compressive strength to tensile strength (Cai 2010) as a material brittleness index (Diederichs et al. 2007; Kahraman et al. 2018; Walton 2014). This parameter is equal to 40.9 for the 0° included angle (no structural influence on failure) specimens presented in this study, whereas other fine-grained sedimentary rocks with similar compressive strengths typically have m_i_ values on the order of 4–7 (Marinos and Hoek 2000). Given that coal is highly brittle relative to its strength, it is expected that the brittle-ductile transition in coal should occur at higher confining stress than for other rocks with similar strength, and therefore at a *σ*_1_/*σ*_3_ ratio below the value of 3.4 expected for most rocks. This is consistent with a *σ*_1_/*σ*_3_ ratio of 2–2.5 as suggested by the testing data presented in this study.

## 6 Prediction of the brittle/ductile transition in coal pillars as a function of W/H ratio

**Fig. 9:**
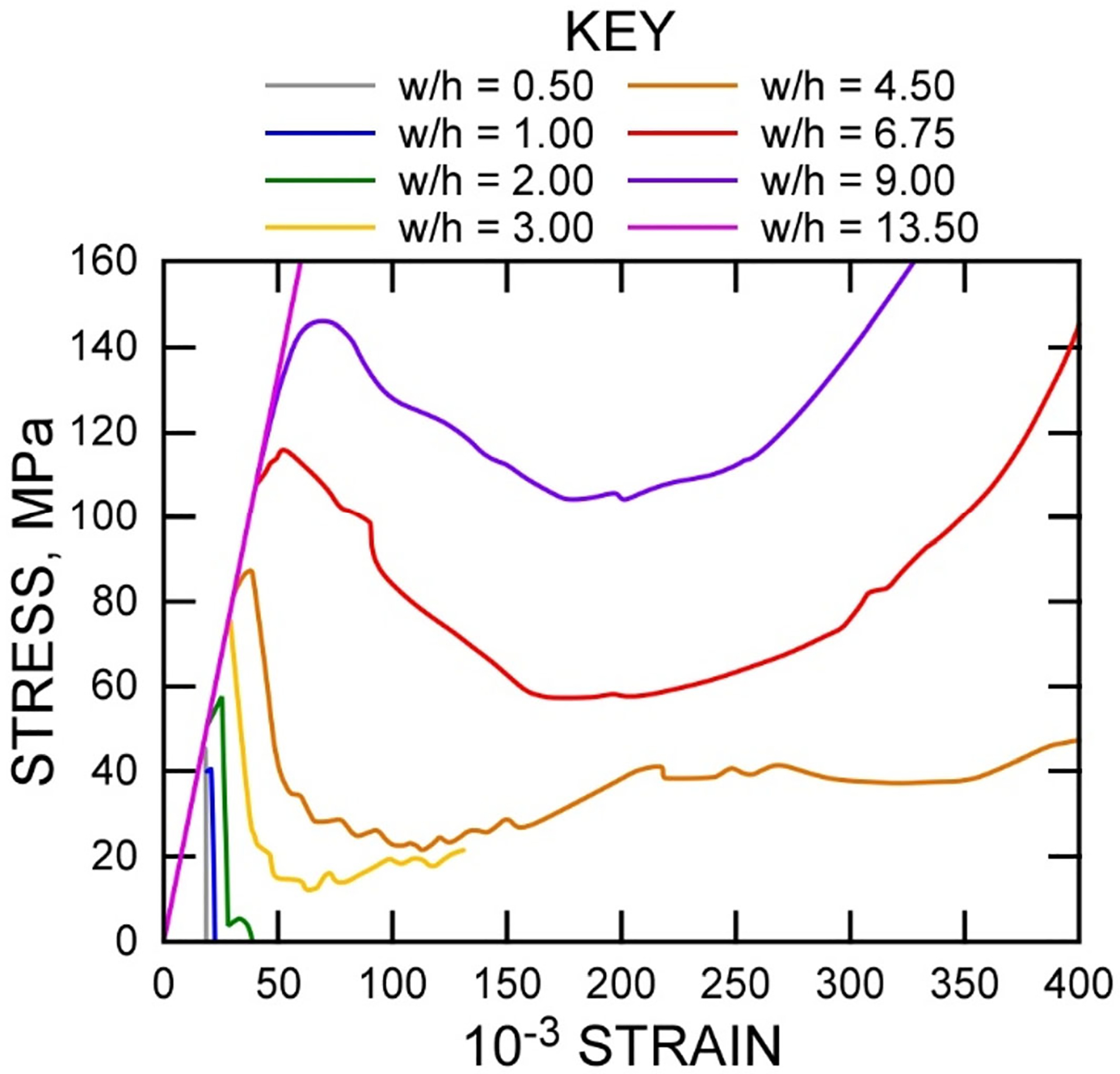
Plots of experimental results of stress versus strain on coal according to various W/H ratios (after (Das 1986, Fig. 3a))

The laboratory results shown in Fig. 5 can help in understanding pillar failure mechanics. Confinement in a pillar is greatly affected by the *W*/*H* ratio of the pillar. Although not considered as a tested parameter in this study, the *W*/*H* ratio of samples has been shown to drastically affect post-peak and residual strength levels, even more so than it affects the peak strength, as shown in Fig. 9 (Das 1986; Özbay 1989). At low *W*/*H* ratios of 0.5 or 1.0, a rock sample may be extremely brittle, losing all strength immediately after attainment of peak strength. With increasing *W*/*H* ratio, the stress–strain curves of uniaxial tests begin to show residual strength, followed by ductile and strain hardening behavior. The degree of hardening is expected to increase with increasing *W*/*H* ratio (Das 1986). Small *W*/*H* ratios have been associated with several massive pillar collapses that occurred in the U.S. (Chase et al. 1994).

Confining stress also has a similar effect in changing a rock sample’s behavior that might be brittle under unconfined conditions to ductile (Cook 1965; Hakami 1988; Kumar et al. 2010; Price 1979; Wawersik and Fairhurst 1970) or even strain hardening (Bawden 2010).

In the case of South African coal mining, the typical pillar *W*/*H* ratio is from 3 to 4 with an average 3 m thick mined seam at a depth of 250 m below the surface. Extensional failure has been observed for mine pillars in South Africa up to *W*/*H* ratios of at least 4 (Mathey and Van der Merwe 2016). The semi-ductile failure mode can occur only in pillars with sufficiently large *W*/*H* ratio, which allows high lateral confinement stresses to be generated within the fractured pillar. While the load bearing capacity of brittle pillars is dominated by the cohesive strength of the material, pseudo-ductile pillars obtain their seemingly unlimited load-bearing capacity from the frictional ductile resistance of fractured material (Mathey and Van der Merwe 2016).

Esterhuizen et al. (Esterhuizen et al. 2010) calibrated numerical coal pillar models against the empirical linear strength formula and measured stress profiles for in situ pillar ribs. The extrapolation of the models to greater *W*/*H* ratios predicts that the brittle-ductile transition occurs in coal pillars having a *W*/*H* ratio of approximately 8. Note that for less brittle rocks, relatively lower confining stresses (and therefore lower *W*/*H* ratios) may be necessary to achieve a ductile pillar stress–strain response (Mortazavi et al. 2009; Sinha and Walton 2018).

Moomivand and Vutukuri (Moomivand and Vutukuri 1996) reported that the coal specimens with a *W*/*H* ratio ranging from 0.25 to 2.0 had brittle behavior and failed completely after yielding, but differences in the yield strength and ultimate strength inside the specimens were increased by an increase in *W*/*H* ratio from 2 to 3.5. The core of pillar specimens behaved in a ductile fashion when the *W*/*H* ratio was greater than 3.5. As the *W*/*H* ratio was greater than or equal to 4, the axial load was increased by more than a factor of two after yielding at the periphery and the insides of a high percentage of the specimens which had ductile behavior were intact (Moomivand and Vutukuri 1996).

The authors presumed that the range of the confining stress considered in the study performed by Kim et al. (2018b) and this study could be substituted by a set of equivalent *W*/*H* ratios of the coal specimens. If there is a meaningful correlation between the confining stress and the *W*/*H* ratios, the result will help us better understand the brittle/ductile transition for in situ pillars.

The effect of an increase in the *W*/*H* ratio on pillar compressive strength, including the increase in lateral stresses in the pillar because of an increase in end constraints, is comparable with the effect of confining stress on a specimen’s axial compressive strength. Using regression formulas, the effect of confining stress on peak strength can be compared to the effect of *W*/*H* of pillar peak strength, meaning an equivalent *W*/*H* value can be derived for each confining stress. It has previously been demonstrated that the relationship between the compressive strength and the *W*/*H* ratio of square prisms when 0.3 ≤ *W*/*H* ratio ≤ 5 is comparable to the relationship between axial compressive stress at failure and confining stress in triaxial tests, but the exponents of the *W*/*H* ratio and confining stress are not equal (Moomivand and Vutukuri 1996). In this section, the exponents of the *W*/*H* ratio and the confining stress showing the relationship between the values are estimated and discussed.

Holland (Holland 1942) performed an extensive laboratory testing program in which coal specimens from different coal seams in West Virginia, USA, were tested at *W*/*H* ratios between 1 and 12. He described the results to be very erratic in general, but demonstrated that a linear or regressive increase in specimen strength up to a *W*/*H* ratio of 8 fit the average data well. The tested coal pillar strength was back-calculated by Mathey and van der Merwe (2016) using the various empirical strength formulae proposed by Bieniawski (1968a, c), Van Heerden (1975) and Wagner (1974). The data fit well to an equation where the strength normalized to UCS was equal to a constant times the square root of *W*/*H* as shown in Eq. (4):

(4) $$\frac{\sigma_{\text{p}}}{{\text{UCS}}_{\text{mean}}}=k\left(\sqrt{\frac{W}{H}}\right)$$

where *σ*_p_ is the pillar strength UCS_mean_ is the average unconfined compressive strength and *k* is a constant.

**Fig. 10:**
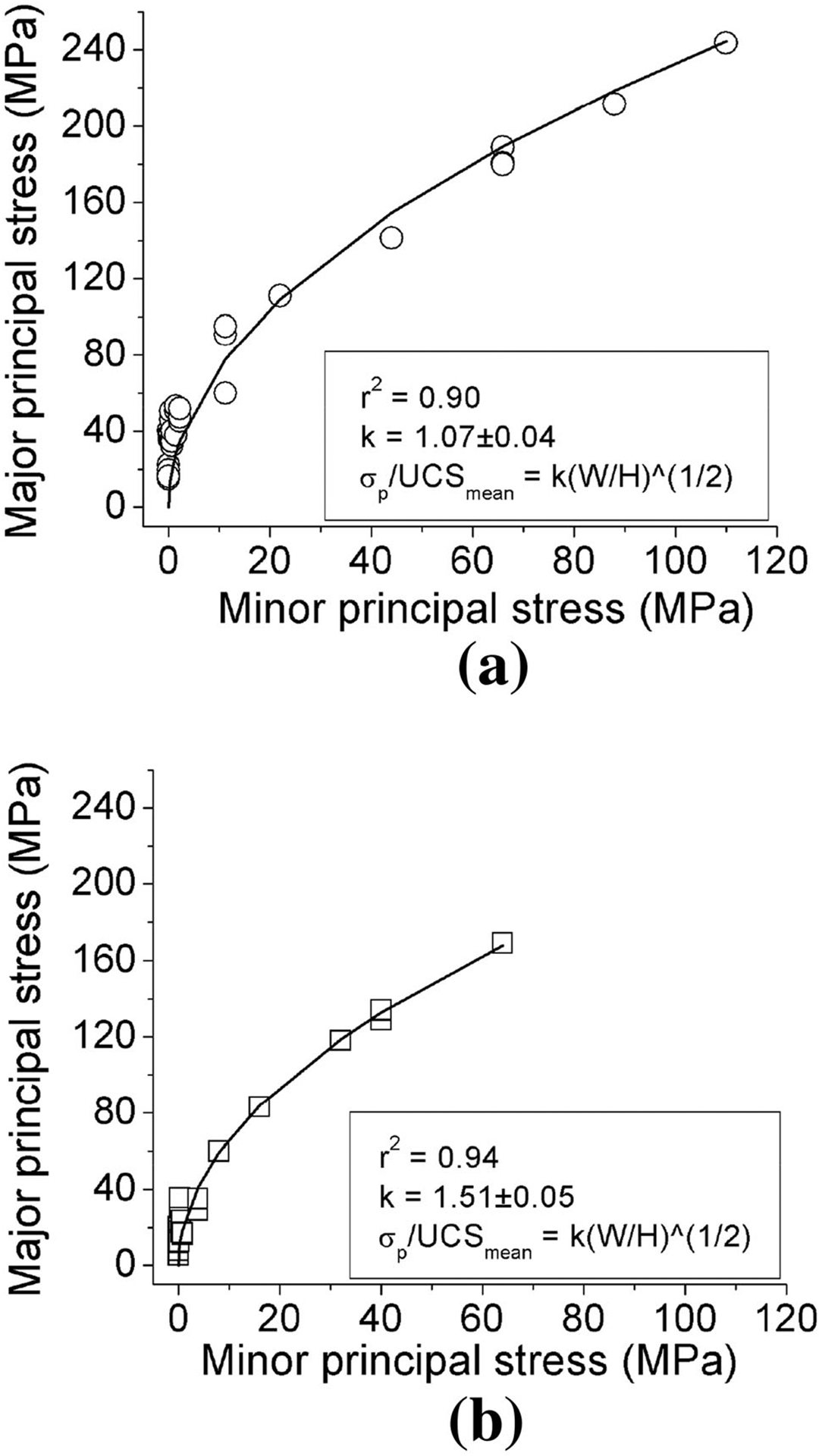
Results of the triaxial compressive tests fitted with the various different empirical methods (**a** 0° of the included angle and **b** 30° of the included angle)

Figure 10 shows the results of all the testing data fitted using the square-root equations of coal pillar strength. It is evident that the data can be represented adequately well (*r*^2^ = 0.90–0.94) by the square-root equation for coal pillar strength.

The authors here adopt a methodology proposed by Moomivand and Vutukuri (1996) showing the relationship between the ratio of compressive strength and *W*/*H* ratio of coal specimens to calculate equivalent exponents of confining stress and *W*/*H* ratio in the equation.

Moomivand and Vutukuri (1996) used the following equations to estimate the exponents of equivalent confining stress that would produce the measured strength in a triaxial compressive test and *W*/*H* ratio:

(5) $$\frac{\sigma_{1}}{\text{UCS}}=1+b^{\prime }{\left(\frac{\sigma_{3}}{\text{UCS}}\right)}^{\beta }$$

(6) σcUCS=A′+B′(WH)α→WH=(σcUCS−A′B′)1α

where *σ*_1_ and *σ*_3_ are the principal stresses, UCS is the unconfined compressive strength, *b*’ and *β* are constants, *σ*_c_ is the compressive strength of pillar specimens, UCS is the unconfined compressive strength, and *A*’, *B*’ and *α* are constants.

**Fig. 11:**
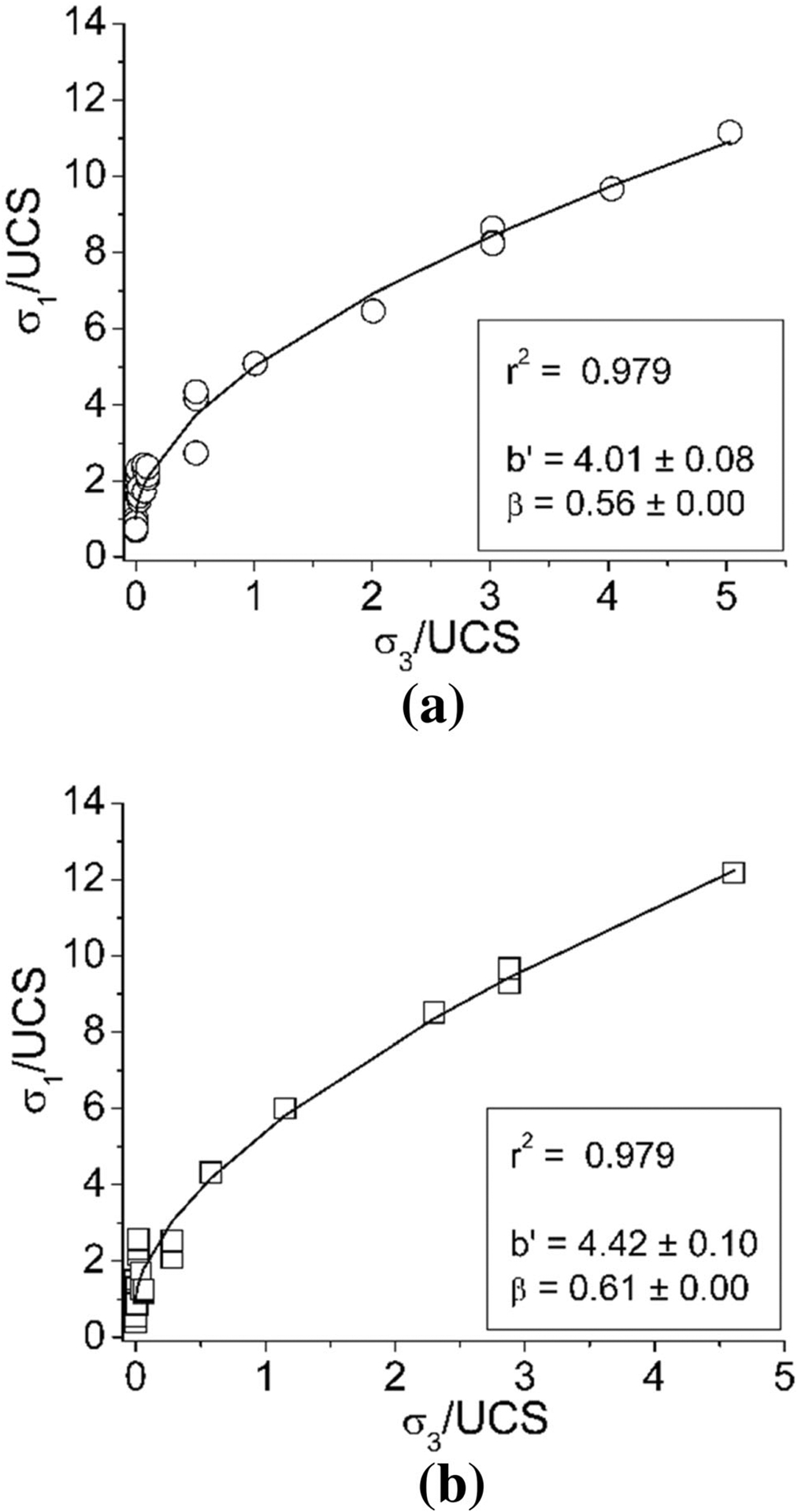
Results of the triaxial compressive tests fitted with the Moomivand & Vutukuri’s approach on the geometry effect of pillar (**a** 0° of the included angle and **b** 30° of the included angle)

**Fig. 12:**
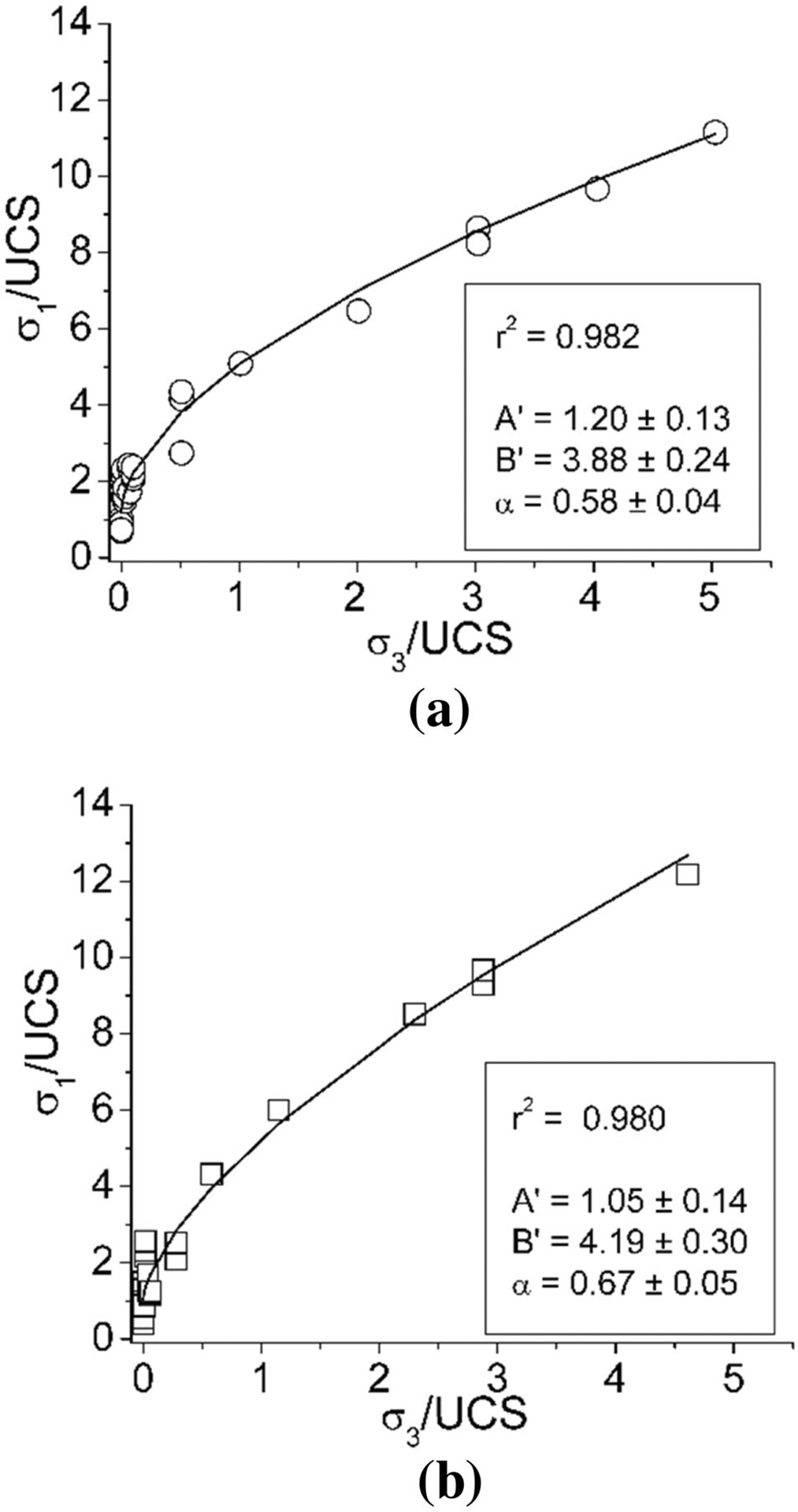
Results of the triaxial compressive tests fitted with the Moomivand & Vutukuri’s approach on the confining effect of pillar (**a** 0° of the included angle and **b** 30° of the included angle)

Using these equations, all the testing data including the results studied by Kim et al. 2018b were analyzed to estimate the exponents of confining stress and *W*/*H* ratio. The results are shown in Figs. 11 and 12.

Mathey and van der Merwe (2016) reported that the evidence presented from field experience, laboratory tests, and numerical and analytical models corroborates the conclusion that a progressive increase in coal pillar strength for *W*/*H* ratios greater than 5 does not exist. Moomivand and Vutukuri (1996) showed that the periphery of a pillar behaves in a brittle fashion for any value of width-to-height ratio and the core of a pillar behaves in a ductile fashion for pillars with *W*/*H* > 3.5.

From the results, one can learn a critical value of *W*/*H* ratio for the Utah coal at which one would expect a transition from brittle to semi-ductile behavior. As shown in Tables 3 and 4, the ratio of the confining stress to the mean UCS in this study is approximately 4 (confining stress of 88 MPa for 0°and 64 MPa for 30°), and the critical equivalent *W*/*H* ratio is around 4.

## 7 Conclusions

In this study, a laboratory investigation using both unconfined and triaxial compressive tests that examine the strength, brittleness, and dilation anisotropy of a Utah coal is presented. Two orientations between the cleat set and axial loading direction in the coal specimen were considered as a testing parameter.

The spalling limit of the coal estimated by the s-shaped brittle failure criterion appears to be dependent on the angle between the cleat and the loading direction, with values of 29 and 41 being observed for included angles of 0° and 30°, respectively. It was also determined that if the confinement level associated with failure mode in the s-shaped criterion are not specifically chosen for characterization of an extremely brittle coal, the spalling limit would be overestimated but the ratio of AUCS to UCS (confined strength) would be underestimated.

Through analysis of post-testing photographs, post-peak stress–strain data, and pre-peak volumetric attributes (crack volumetric strain reversal and dilation angle mobilization), it was determined that for the 0° included angle specimens, the brittle-ductile transition initiates around *σ*_3_ = 88 MPa; for the 30° included angle specimens, the brittle-ductile transition initiates around *σ*_3_ = 40–64 MPa. These values are both much higher than would be expected based on conventional models for the brittle-ductile transition in rock (Mogi 1966), and this was attributed to the anomalously high brittleness to strength ratio of the rock considered in this study.

Since the coal showed that the mechanical characteristics are strongly anisotropic and confinement-dependent, a better characterization of coal for the analysis and design of excavations and pillars is an important step toward improving miner safety with respect to stability of underground workplaces and the prevention of fatalities. Towards this goal, a previously proposed relationship was used to convert the confining stresses considered in the laboratory tests into equivalent pillar *W*/*H* ratios. Based on this, it was determined that in the coal considered in this study, pillars would transition from brittle to ductile behavior at *W*/*H* ratios of approximately 4 for the Utah coal specimens. The critical value of W/H ratio for the Utah coal may need to be investigated further by considering an in situ condition for future work.

Although the focus of this study was specifically on the Utah coal, the findings can be compared with those from previously published studies to better understand the nature of the brittle-ductile transition in rocks. Specifically, the ratio of confining stress to UCS at which the brittle-ductile transition will tend to higher for more brittle rocks (i.e. rocks with higher *m*_i_ values). In the context of pillar performance in underground mines, this means that while increased *W*/*H* (and the corresponding increase in confining stress) will tend to lead to less brittle overall pillar behavior in a specific rock type, the *W*/*H* ratio at which pillar behavior transitions from brittle to ductile will tend to be higher for pillars composed of rock which is more brittle.

## Figures and Tables

**Table 1:** Summary of the average mechanical properties

The modified ratio of confining stress to UCS after Kaiser and Kim (2008a, 2015)	Anticipated failure mode
1%−50%	Extensional
50%−100%	Transitional
> 100%	Shear

**Table 2:** Re-calculated spalling limits and the ratios of apparent UCS to UCS using the modified failure mechanism transition levels

Cleat angle (°)	AUCS/UCS	*k*_sp_
0	3.6	29.1
30	3.3	40.8

**Table 3:** Relationship between ratio of strength and *W*/*H* ratio of 0 degrees sample

*σ*_3_ (MPa)	*σ*_1_ (MPa)	*σ*_3_/UCS	*σ*_1_/UCS	*W/H*
22	104.3	1.0	5.1	1.0
44	143.4	2.0	6.5	1.7
66	174.4	3.0	8.6	3.1
66	174.4	3.0	8.3	2.8
66	174.4	3.0	8.2	2.8
88	201.0	4.0	9.7	3.8
110	224.9	5.0	11.2	5.1

**Table 4:** Relationship between ratio of strength and *W*/*H* ratio of 30 degrees sample

*σ*_3_ (MPa)	*σ*_1_ (MPa)	*σ*_3_/UCS	*σ*_1_/UCS	*W/H*
16	80.1	1.1	6.0	1.3
32	114.9	2.3	8.5	2.4
40	129.7	2.9	9.3	2.8
40	129.7	2.9	9.7	2.9
40	129.7	2.9	9.7	2.9
64	168.1	4.6	12.2	4.3
